# Erfolgreiche Wiedereingliederung und ökonomisch sinnvoll: Die Einstiegsprämie als neue Maßnahme für Return to Work

**DOI:** 10.1055/a-2724-3608

**Published:** 2025-12-09

**Authors:** Andrea Bökel, Tatjana Levin, Christian Sturm, Christoph Egen, Anne Ostermann

**Affiliations:** 1 Klinik für Rehabilitations- und Sportmedizin, Medizinische Hochschule Hannover

**Keywords:** Prämie, Return-to-work, Teilhabe am Arbeitsleben, Bonus, return to work, participation in working life

## Abstract

**Ziel:**

Die Einstiegsprämie (ESP) ist eine innovative Leistung zur Teilhabe am Arbeitsleben, die im Rahmen des rehapro-Förderprogramms erprobt wurde. Ihr Hauptziel ist, die rasche und selbstvermittelte Wiedereingliederung von Menschen mit langfristigen gesundheitlichen Beeinträchtigungen in den Arbeitsmarkt. Die ESP wird als Alternative zum Eingliederungszuschuss (EGZ) angeboten und bietet den Beschäftigten eine monatliche Unterstützung von bis zu 400 Euro über einen Zeitraum von sechs Monaten sowie eine einmalige Erfolgsprämie nach zwölf Monaten im Arbeitsverhältnis. Die Maßnahme soll zur Beschäftigungsaufnahme motivieren, den Übergang in das Erwerbsleben erleichtern und die Chancen auf gesellschaftliche Teilhabe verbessern.

**Methodik:**

In der Kohortenstudie wurden Reha-Statistik-Daten (RSD) von Versicherten der Rentenversicherungsträger Mitteldeutschland und Braunschweig-Hannover, die die ESP bzw. den EGZ bezogen, ausgewertet. Der Hauptzielparameter ist die Wiedereingliederungsquote sechs, 13 und 15 Monate nach Maßnahmenbeginn und ein Kostenvergleich. Sekundäre Parameter wie u. a. Tätigkeitsbranchen wurden mittels deskriptiver Berechnungen für beide Gruppen analysiert. Des Weiteren erfolgte eine binäre logistische Regressionsanalyse zu Untersuchung von Prädiktoren einer 15-monatigen Erwerbstätigkeit.

**Ergebnisse:**

Im Interventionszeitraum bezogen 485 Personen die ESP. Die Analyse der RSD ergibt für die Gruppe der ESP-Beziehenden eine höhere Beschäftigungsquote für die Zeiträume von 6 Monate, 13 Monate und 15 Monaten nach Maßnahmenbeginn (91,3% für 6 Monate, 83,7% für 13 Monate und 81,2% für 15 Monate nach Maßnahmenbeginn). Die Beschäftigungsquote der EGZ-Gruppe lag bei 89,3% nach 6 Monaten, bei 68,7% nach 13 Monaten und bei 63,4% bei 15 Monaten nach Maßnahmenbeginn. ESP-Beziehende hatten eine 2,4-fach erhöhte Chance 15 Monate Erwerbstätigkeit zu erreichen, als EGZ-Beziehende. Die Maßnahmenkosten pro Fall sind in der EGZ-Gruppe 2,8-fach höher als in der ESP-Gruppe.

**Schlussfolgerung:**

Aus der mit der ESP erreichten Wiedereingliederungsquote und der ökonomischen Analyse lässt sich eine positive Bilanz für das Modellprojekt ziehen. Die Leistung erreicht eine Zielgruppe, die gesundheitlich langfristig eingeschränkt ist und zum Teil langzeitarbeitslos war. **Kernbotschaft** : Die Einstiegsprämie ist eine nicht zweckgebundene Leistung an Arbeitnehmende. Im Vergleich zu Zahlungen an Arbeitgebende erzielt sie als Wahlalternative eine bessere Eingliederungsquote bei niedrigeren Kosten als der EGZ.

## Abstract (en)

**Objective:**

The entry bonus (ESP) is an innovative benefit designed to promote participation in working life, which has been trialled as part of the rehapro support programme. Its main objective is to facilitate the rapid and self-directed reintegration of people with long-term health impairments into the labour market. The ESP is offered as an alternative to the integration subsidy (EGZ) and provides employees with monthly support of up to €400 over a period of six months, as well as a one-time success bonus after twelve months of employment. The measure is intended to motivate people to take up employment, facilitate their transition into working life and improve their chances of participating in social life.

**Methodology:**

In the cohort study, rehabilitation statistics data (RSD) from insured persons of the pension insurance institutions Mitteldeutschland and Braunschweig-Hannover who received the ESP or EGZ were evaluated. The main target parameter was the reintegration rate six, 13 and 15 months after the start of the measure and a cost comparison. Secondary parameters such as the sectors of employment were analysed for both groups using descriptive analyses. In addition, a binary logistic regression analysis was performed to investigate predictors of 15 months of employment.

**Results:**

During the intervention period, 485 persons received ESP. The RSD analysis showed a higher employment rate for the group receiving ESP for the periods of 6 months, 13 months and 15 months after the start of the measure (91.3% for 6 months, 83.7% for 13 months and 81.2% for 15 months after the start of the measure). The employment rate for the EGZ group was 89.3% after 6 months, 68.7% after 13 months and 63.4% after 15 months after the start of the programme. ESP recipients had 2.4 times higher odds to be in employment after 15 months than EGZ recipients. The measure costs per case were 2.8 times higher in the EGZ group than in the ESP group.

**Conclusion:**

Reintegration rate and economic analysis of the ESP show a positive outcome of the pilot project. The benefit reaches a target group that has long-term health restrictions and, in some cases, has been unemployed for a long-term period. **Key message** : The entry bonus is a non-purpose-tied benefit for employees. Compared to payments to employers, ESP is an alternative option and achieves a better integration rate at lower costs than the EGZ.

## Einleitung

Die nachhaltige Wiedereingliederung in das Erwerbsleben von Menschen mit chronischen Erkrankungen oder Behinderungen ist ein zentrales Ziel der Deutschen Rentenversicherung (DRV). Neben der finanziellen Absicherung der Betroffenen hat die Erwerbstätigkeit eine weitreichende Bedeutung für das Individuum und die Gesellschaft. Arbeit bedeutet soziale Interaktion, sie trägt zur Identitäts- und Sinnstiftung bei, strukturiert den Alltag und fördert die gesellschaftliche Teilhabe 1 2 . Der Erhalt und die Wiederherstellung der Erwerbsfähigkeit sind daher essenzielle Bestandteile der Rehabilitation (§ 42 Absatz 1 Satz 1 SGB) 3 4 , die sowohl aus gesundheitspolitischer als auch aus sozialökonomischer Perspektive von großer Relevanz sind, da sie die Teilhabe am Arbeitsleben von Menschen mit Behinderungen oder gesundheitlichen Einschränkungen fördert. Sie trägt dazu bei, die Erwerbsfähigkeit zu erhalten oder wiederherzustellen und kann so die soziale Integration fördern und die Lebensqualität verbessern 4 .

Trotz bestehender Rehabilitations- und Eingliederungsmaßnahmen stehen viele Personen, insbesondere nach längeren Krankheitsphasen, vor erheblichen Herausforderungen bei der Rückkehr in den Arbeitsmarkt. Personen, die z. B. nach einer Erwerbsminderungsrente wieder in das Erwerbsleben einsteigen, äußern einerseits Sorgen und Ängste, wie Versagensangst oder das Empfinden von viel Stress. Andererseits ist die Rückkehr ins Erwerbsleben verbunden mit Hoffnung auf Erfolgserleben, soziale Kontakte und Anerkennung. Neben gesundheitlichen und strukturellen Hürden spielen auch finanzielle Aspekte eine Rolle. Viele Betroffene erleben durch Einkommenseinbußen eine finanzielle Unsicherheit, die den Wiedereinstieg in das Erwerbsleben zusätzlich erschwert 5 . Eine zentrale Frage in diesem Kontext ist, wie finanzielle Anreize genutzt werden können, um den Wiedereinstieg und den langfristigen Verbleib im Arbeitsmarkt zu fördern.

Bezüglich der Wirkung finanzieller Anreize auf das Verhalten von Erwerbstätigen zeigen Studien, dass monetäre Belohnungen eine leistungssteigernde Wirkung haben können und zu einer stärkeren Zielorientierung beitragen 6 7 . Gleichzeitig wird darauf hingewiesen, dass sie allein nicht ausreichen, um die intrinsische Motivation nachhaltig zu erhöhen 8 . Studien, die Prämienzahlungen im Zusammenhang mit einem Return-to-Work bei gesundheitlich eingeschränkten Personen untersuchen liegen bislang nicht vor.

Vor diesem Hintergrund wurde im Rahmen der Förderinitiative rehapro das Projekt Einstiegsprämie (ESP) durchgeführt. Ziel des Projekts war es, die Motivation zur Aufnahme einer sozialversicherungspflichtigen Beschäftigung durch eine zeitlich befristete, nicht zweckgebundene Prämienzahlung zu fördern. Die Maßnahme wurde von der DRV Mitteldeutschland (MD) und der DRV Braunschweig-Hannover (BS-H) initiiert und in Kooperation mit der Klinik für Rehabilitations- und Sportmedizin der Medizinischen Hochschule Hannover wissenschaftlich evaluiert. Die ESP wurde als Wahlalternative zum etablierten Eingliederungszuschuss (EGZ) angeboten. Während beim EGZ finanzielle Mittel an Arbeitgebende ausgezahlt werden, setzt das ESP-Modell direkt bei den Versicherten an.

Die vorliegende Studie hat das Ziel die Wiedereingliederungsquoten und die mit den Maßnahmen einhergehenden Kosten zwischen den beiden Gruppen zu vergleichen.

## Methodik

Die kontrollierte prospektive Interventionsstudie erfolgte im Einzugsgebiet der DRVen MD und BS-H im Interventionszeitraum von Januar 2020 bis Oktober 2023. Die Evaluation erfolgte durch Interviews, eine Fragebogenerhebung und die hier berichtete Analyse der Routinedaten der DRV. Eine fallweise Verknüpfung mit den Daten der Interviewanalyse und der Fragebogenerhebung war aus datenschutzrechtlichen Gründen nicht möglich. Das Studienprotokoll wurde von der Ethikkommission der Medizinischen Hochschule Hannover geprüft (NR. 9533_BO_K_2020). Die prospektive Studienregistrierung erfolgte im Deutschen Register Klinischer Studien (DRKS00033870).

### Beschreibung der Intervention

Die Einstiegsprämie wurde an Personen gezahlt, die sich eigenständig eine sozialversicherungspflichtige Beschäftigung suchten. Vollzeitbeschäftigte erhielten dabei eine monatliche Unterstützung von 400 Euro für die ersten 6 Monate, Teilzeitbeschäftigte anteilig weniger. Zusätzlich wurde auf Antrag der Teilnehmenden eine einmalige Prämie von 600 Euro ausgezahlt, wenn das Arbeitsverhältnis über einen Zeitraum von mindestens 12 Monaten fortbestand. Ziel war es, zur Arbeitsaufnahme zu motivieren, finanzielle Barrieren zu reduzieren und dadurch die nachhaltige berufliche Integration zu erleichtern.

### Stichprobe und Daten

In die Studie konnten alle Personen eingeschlossen werden, die einen EGZ oder eine ESP im Interventionszeitraum bezogen. Wurde ein Bescheid zur Berechtigung erteilt, wurde über beide Maßnahmen schriftlich informiert und die Versicherten konnten sich für eine der beiden Leistungen freiwillig entscheiden. Die DRV BS-H hat mit allen Versicherten zusätzlich persönliche Beratungs- und Informationsgespräche durchgeführt. Die DRV MD führte partiell telefonische Beratungs- und Informationsgespräche durch. Versicherte, die ihr schriftliches Einverständnis zur Studienteilnahme erklärten, wurden in die Studie eingeschlossen. Die Routinedaten der Rentenversicherung wurden anonymisiert für Analysezwecke zur Verfügung gestellt und bedürfen keiner besonderen Einwilligung.

Die Analyse der Routinedaten untersuchte die Unterschiede zwischen ESP-Beziehenden und EGZ-Beziehenden. Die Datenbasis bestand aus Datensätzen der Reha-Statistik-Datenbasis (RSD), den jeweiligen Zahldaten in Euro und der Statistik 96 (SY96), welche Informationen über bewilligte und durchgeführte Leistungen zur Teilhabe am Arbeitsleben enthält. Das primäre Zielkriterium war die sozialversicherungspflichtige Beschäftigungsdauer nach Beginn der Maßnahmen ESP bzw. EGZ. Sekundäre Zielkriterien waren die Maßnahmenkosten pro Fall, Leistungsabbrüche, die Stellung im Beruf, die Art der Arbeit bei Antragsstellung, die Beitragszeiten 12 Monate vor dem individuellen Maßnahmenbeginn und die Branchen, in denen die Teilnehmenden tätig sind.

Die pseudonymisierten Datensätze zur Reha-Statistik-Datenbasis sowie Systemdaten (SY96) und Zahldaten wurden von den DRVen MD und BS-H im August bzw. November 2024 zur Verfügung gestellt. Die Daten wurden im Dateiformat Excel (DRV MD) bzw. im Dateiformat Textdatei (DRV BS-H) übermittelt und anschließend im Rahmen des Datenmanagements bezüglich ihrer Vollständigkeit und Plausibilität geprüft. Auffälligkeiten und nicht plausible Daten wurden mit den zuständigen Mitarbeitenden der DRVen geklärt. Daraufhin wurden die Datensätze in die Statistiksoftware SPSS Statistics (IBM, Version 29.0.1.0) importiert und zusammengeführt.

Zur Ermittlung der Wiedereingliederungsquoten wurde als Zielkriterium die Dauer der sozialversicherungspflichtigen Beschäftigung nach dem Maßnahmenbeginn in der Interventions- versus Kontrollgruppe verglichen. Hauptzielparameter war eine mind. 15-monatige Beschäftigung. Die Erwerbstätigkeit wurde daher in einem Zeitraum von bis zu 15 Monaten nach Maßnahmenbeginn erfasst. Zur Evaluation wurden die Maßnahmenkosten pro Fall in Euro analysiert und damit der finanzielle Aufwand in den Gruppen der ESP und EGZ vergleichend gegenübergestellt. Sekundäres Zielkriterium waren die Beitragszeiten 12 Monate vor Maßnahmenbeginn. Anhand derer wurde beschrieben, ob die Zielgruppe vor der Maßnahme einer versicherungspflichtigen Beschäftigung nachgegangen ist oder ggf. Arbeitslosengeld oder andere Leistungen erhalten hat. Um Abbrüche der Maßnahme zu beschreiben wurde das Ergebnis der Leistung analysiert. Die Tabelle S1 zeigt einen Überblick über alle Zielkriterien, die Datenquellen und die verwendeten statistischen Methoden.

### Statistische Analysen

Stichprobenkennwerte wurden zunächst deskriptiv analysiert. Die Normalverteilung wurde anhand des Shapiro-Wilk-Tests überprüft. Zur Prüfung der Unterschiede wurden je nach Skalenniveau Chi-Quadrat- oder T-Tests bei nicht-gegebener Normalverteilung der Test nach Mann-Whitney U durchgeführt. Zudem wurde eine binäre logistische Regressionsanalyse durchgeführt. Dafür wurde eine Multikollinearitätsanalyse durchgeführt. In einem ersten Schritt wurden alle potenziellen unabhängigen Variablen (Gruppenzugehörigkeit ESP/EGZ, Geschlecht, Alter, RV Trägerin, Stellung im Beruf, Branche) in eine explorative Regressionsanalyse inkludiert. Nur jene Prädiktoren mit p<0,05 wurden in einem zweiten Schritt in die finale binäre Regressionsanalyse mit der Methode schrittweiser Einschluss inkludiert. Für alle Ergebnisse wurde das Signifikanzniveau p<0,05 festgelegt. Alle Auswertungen erfolgen durch eine Mitarbeiterin der Klinik für Rehabilitations- und Sportmedizin der Medizinischen Hochschule Hannover unter Verwendung von SPSS Statistics (IBM, Version 29.0.2.0).

## Ergebnisse

### Soziodemografie

Aus dem Zeitraum vom 01.04.2020 bis 31.03.2023 lagen Daten von insgesamt 485 Personen vor, die eine ESP erhalten haben. Die Kontrollgruppe erhielt den EGZ im Zeitraum 01.10.2020-01.10.2023. Das durchschnittliche Alter der Teilnehmenden lag bei 49 Jahren. 27,4% waren weiblich ( Tab. 1 ).

**Table:** **Tab. 1** Demografie der Gesamtkohorte, der Kohorte der DRV Mitteldeutschland und der Kohorte DRV Braunschweig-Hannover.

Stichprobenmerkmale	Gesamtkohorte			DRV Mitteldeutschland (MD)			DRV Braunschweig-Hannover (BS-H)		
	Gesamt	ESP	EGZ	Gesamt	ESP	EGZ	Gesamt	ESP	EGZ
Anzahl (n)	2.204	485	1.719	889	224	665	1.315	261	1.054
Alter in Jahren Mittelwert, Median (Std.-Abw.; Min; Max)	49,32; 51 (8,393; 23; 65)	48,19; 49 (8,362; 24; 65)	49,64; 51 (8,376; 23; 65)	49,89; 51 (8,081; 25; 64)	49,33; 51 (8,182;28; 64)	50,08; 51 (8,043; 25; 64)	48,94; 50 (8,579; 23; 65)	47,21; 47 (8,406; 24; 65)	49,36; 51 (8,572; 23;65)
Geschlecht männlich n (%)	1.600 (72,6)	315 (64,9)	1.285 (74,8)	615 (69,2)	150 (67,0)	465 (69,9)	985 (74,9)	165 (63,2)	820 (77,8)
weiblich n (%)	604 (27,4)	170 (35,1)	434 (25,2)	274 (30,8)	74 (33,0)	200 (30,1)	330 (25,1)	96 (36,8)	234 (22,2)
Altersunterschiede nach Geschlecht und Gruppenzugehörigkeit M (Std.-Abw.)									
	**Gesamtkohorte**			**DRV Mitteldeutschland (MD)**			**DRV Braunschweig-Hannover (BS-H)**		
	männlich	weiblich	p	männlich	weiblich	p	männlich	weiblich	p
ESP	48,81 (8,209)	47,02 (8,540)	,024	49,61 (8,278)	48,76 (8,010)	,446	48,09 (8,104)	45,69 (8,735)	,026
EGZ	49,92 (8,231)	48,79 (8,748)	,015	50,01 (8,149)	50,24 (7,809)	,737	49,88 (8,281)	47,56 (9,319)	<,001

Sowohl in der Kohorte der ESP-Beziehenden als auch in der Gruppe der EGZ-Beziehenden waren die Männer signifikant älter als die Frauen ( Tab. 1 ).

In der Kohorte der DRV MD unterschieden sich sowohl in der Gruppe der ESP-Beziehenden als auch in der Gruppe der EGZ-Beziehenden Frauen und Männer nicht im Hinblick auf das Durchschnittsalter. In der Kohorte der DRV BS-H hingegen unterschieden sich sowohl in der Gruppe der ESP-Beziehenden als auch in der Gruppe der EGZ-Beziehenden Frauen und Männer signifikant im Hinblick auf das Durchschnittsalter ( Tab. 1 ).

### Merkmale der Tätigkeiten vor Maßnahmenbeginn

Ungefähr ein Drittel der Teilnehmenden waren in der letzten Tätigkeit vor Maßnahmenbeginn in der Rohstoffgewinnung, Produktion und Fertigung tätig, gefolgt von der Branche Verkehr, Logistik, Schutz und Sicherheit und der Branche Bau, Architektur, Vermessung, Gebäudetechnik (Tab. S2). Die Kohorte besteht zu ca. der Hälfte aus Facharbeiter:innen und zu einem Fünftel aus Angestellten. In DRV MD ist der relative Anteil an Facharbeiter:innen höher als in DRV BS-H. Gleichzeitig ist in der DRV BS-H der relative Anteil der Angestellten im Vergleich zu DRV MD größer (Tab. S3). Bei Antragsstellung gaben 46,9% der Teilnehmenden in der DRV MD und 13,5% der Teilnehmenden in DRV BS-H an arbeitslos gemeldet zu sein. Ganztagsarbeit leisteten zu diesem Zeitpunkt 46,3% in der DRV MD und 76,9% in der DRV BS-H (Tab. S4).

### Beitragszeiten vor Maßnahmenbeginn

Im Zeitraum von 12 Monaten vor Maßnahmenbeginn zahlten 19,46% (n=429) der Teilnehmenden durchschnittlich 5,08 Monate (SD=3,67) keine freiwilligen oder Pflichtbeiträge. Keine Person zahlte freiwillige Beiträge (n=2.204). In den 12 Monaten vor Maßnahmenbeginn zahlten 40,43% (n=891) der Teilnehmenden im Durchschnitt für 4,23 Monate (SD=3,467) Pflichtbeiträge aufgrund versicherungspflichtiger Beschäftigung. Davon zahlten 22,1% für einen bis drei Monate Beiträge, während weitere 8,7% (n=192) vier bis sechs Monate Beiträge zahlten. Weitere 4,7% (n=104) zahlten für sieben bis neun Monate Pflichtbeiträge und 5% (n=108) zahlten zehn bis zwölf Monate Pflichtbeiträge aufgrund sozialversicherungspflichtiger Beschäftigung vor Maßnahmenbeginn. Keine Pflichtbeiträge aufgrund versicherungspflichtiger Beschäftigung zahlten 59,5% (n=1.312) der Teilnehmenden in den 12 Monaten vor Maßnahmenbeginn. Das bedeutet, dass ca. 2/3 der Teilnehmenden min. ein Jahr lang arbeitslos waren, bevor sie die Leistung bezogen. Ein Drittel war im Jahr vor Maßnahmenbeginn im Schnitt ca. 8 Monate arbeitslos.

Die Pflichtbeitragszeit wegen sonstigem Leistungsempfang nach § 3 Nr. 3 SGB VI, § 4 Abs. 3 SGB VI, also Krankengeld, Übergangsgeld oder Arbeitslosengeld lag bei 76,15% (n=2.205) der Teilnehmenden durchschnittlich bei 7,44 Monaten (SD=3,852). Darunter waren 12,1%, die über die Dauer von 12 Monaten diese Pflichtbeiträge zahlten.

Geringfügige Beschäftigung (mit und ohne Befreiung von der Versicherungspflicht bzw. mit und ohne Verzicht auf die Versicherungsfreiheit (bis 31.12.2012); auch im Privathaushalt) (§ 8 Abs. 1Nr. 1 SGB IV, § 8a SGB IV) lag in den 12 Monaten vor Maßnahmenbeginn bei 0,73% (n=15) der Teilnehmenden für durchschnittlich 4,53 Monate (SD=3,523) vor.

Abbrüche der Leistung fanden in der Kohorte der ESP-Beziehenden bei 1,05% und in der Kohorte der EGZ-Beziehenden bei 2,96% der Teilnehmenden statt.

### Beschäftigungsdauer nach Maßnahmenbeginn

Die Beschäftigungsdauer wurde gestaffelt zu drei Messzeitpunkten betrachtet: 6 Monate, 13 Monate und 15 Monate nach Beginn der Maßnahme ESP bzw. EGZ. Eine mindestens 15-monatige Erwerbstätigkeit erreichten signifikant häufiger ESP-Beziehende (81,2%) im Vergleich zu EGZ-Beziehenden (63,4%) ( Tab. 2 ).

**Table:** **Tab. 2** Beschäftigungsdauer nach Maßnahmenbeginn der Gesamtkohorte, der Kohorte der ESP-Beziehenden und der Kohorte der EGZ-Beziehenden.

Beschäftigungsdauer nach Maßnahmenbeginn	DRV Mitteldeutschland U=58227,000, z=−3,733, p<0,001; r=0,13				DRV Braunschweig-Hannover U=81181,500, z=−6,914, p<0,001; r=0,21				Gesamtkohorte U=278399,000, z=−7,701, p<0,001; r=0,16			
	ESP (n=224)		EGZ (n=665)		ESP (n=261)		EGZ (n=1054)		ESP (n=485)		EGZ (n=1719)	
	*n*	*%*	*n*	*%*	*n*	*%*	*n*	*%*	*n*	*%*	*n*	*%*
**Mindestens 6 Monate**	210	93,8	638	95,9	233	89,3	897	85,1	443	91,3	1535	89,3
**Mindestens 13 Monate**	188	83,9	502	75,5	218	83,5	679	64,4	406	83,7	1181	68,7
**Mindestens 15 Monate**	179	79,9	462	69,5	215	82,4	627	59,5	394	81,2	1089	63,4

### Maßnahmenkosten

Die Kosten pro versicherte Person beliefen sich in der Gruppe der ESP-Beziehenden im Mittelwert auf 2.578,83 € und sind in der Gruppe der EGZ-Beziehenden mit einem Mittelwert von 7.194,42 € 2,8-fach höher ( Tab. 3 ). Die Erfolgsprämie wurde an 65,5% (n=171) der ESP-Beziehenden in der DRV BS-H und an 68,6% (n=153) der ESP-Beziehenden in der DRV MD ausgezahlt.

**Table:** **Tab. 3** Maßnahmenkosten der Kohorte der ESP-Beziehenden und der Kohorte der EGZ-Beziehenden in der Gesamtkohorte und nach den Trägerinnen DRV MD und DRV BS-H aufgeteilt.

Maßnahmenkosten	Gesamtkohorte		DRV Mitteldeutschland (MD)		DRV Braunschweig-Hannover (BS-H)	
	EGZ (n=1.698)	ESP (n=479)	EGZ (n=662)	ESP (n=222)	EGZ (n=1.036)	ESP (n=257)
Summe Gesamtkosten	12.216.122,64 €	1.235.258,60 €	5.185.804,69 €	589.300,00 €	7.030.317,95 €	645.958,60 €
Mittelwert (Kosten pro Fall)	7194,42 €	2.578,83 €	7.833,54 €	2.654,51 €	6.786,02 €	2.513,46 €
Median	6222,42 €	3.000,00 €	7.053,01 €	3000,00 €	6000,00 €	3.000,00 €
SD	4602,41 €	603,63 €	4.370,12 €	525,51 €	4.701,65 €	657,79 €
Minimum	93,00 €	200,00 €	93,00 €	300,00 €	500,00 €	200,00 €
Maximum	60.000,00 €	3.000,00 €	33.670,00 €	3.000,00 €	60.000,00 €	3.000,00 €
Spannweite	59.907,00 €	2.800,00 €	33.577,00 €	2.700,00 €	59.500,00 €	2.800,00 €

### Zusammenhang zwischen 15-monatiger Erwerbstätigkeit und unabhängigen Variablen

Die binäre logistische Regressionsanalyse untersuchte, welche unabhängigen Variablen die Wahrscheinlichkeit des Erreichens von mindestens 15 Monaten Erwerbstätigkeit nach Maßnahmenbeginn beeinflussten. Es wurde zunächst ein exploratives Regressionsmodel mit den unabhängigen Variablen Geschlecht, Branche, Stellung im Beruf, Rentenversicherungsträgerin, Alter und Gruppenzugehörigkeit zur ESP oder EGZ berechnet, wobei die drei letztgenannten unabhängigen Variablen signifikant waren und in das finale Regressionsmodel inkludiert wurden. Das finale Modell mit der Methode Einschluss war als Ganzes signifikant (Chi-Quadrat(2) 73,207, p<0,001). Die Korrelationen zwischen den Prädiktoren war gering (r<0,059), was darauf hindeutet, dass Multikollinearität die Analyse nicht konfundiert hat 9 . Das Nagelkerke R ^2^ lag bei 0,046. ESP-Beziehende hatten eine 2,4-fach erhöhte Chance 15 Monate Erwerbstätigkeit zu erreichen, als EGZ-Beziehende. Mit jedem Lebensjahr sanken die Chancen 15 Monate Erwerbstätigkeit nach Maßnahmenbeginn zu erreichen um 0,02%. Versicherte der DRV BS-H hatten eine 1,4-fach höhere Chance die 15-monatige Erwerbstätigkeit zu erreichen als Versicherte der DRV MD (Tab. S5). Cohens F ^2^ betrug 0,05, was nach Cohen einem schwachen Effekt entspricht 10 .

## Diskussion

Die Ausgangsfrage dieser Studie war, ob mit einer neuartigen Leistung in Form einer nicht zweckgebundenen Direktzahlung eine vergleichbare Wiedereingliederungsquote erreicht werden kann wie mit etablierten Zahlungen an Arbeitgebende. Die Ergebnisse zeigen, dass die Return-to-Work-Quoten beider Leistungen nach sechs Monaten vergleichbar sind und die Personen mit der ESP langfristig signifikant länger im Erwerbsleben bleiben als Personen mit EGZ-Bezug ( Tab. 2 ). Die ökonomische Analyse zeigt, dass für die guten Effekte der ESP nur 35,8% der Mittel aufgewendet werden mussten wie für die Regelleistung EGZ ( Tab. 3 ). Da es sich um eine Wahlleistung handelt, sind die Ergebnisse nicht so zu interpretieren, dass die ESP den EGZ ersetzen kann, jedoch für einen großen Teil der Zielgruppe die langfristig geeignetere Maßnahme zu sein scheint und für den Sozialversicherungsträger in vielen Fällen einen wesentlich effizienteren Mitteleinsatz bedeutet. Parallel durchgeführte Fragebogen- und Interviewanalysen zeigten außerdem eine hohe Akzeptanz und Zufriedenheit der Versicherten. Diese Daten werden an anderer Stelle publiziert 11 .

Es zeigte sich, dass Personen, die die ESP gewählt hatten, häufiger 15 Monate Erwerbstätigkeit bei geringeren Kosten erreichten, als die Vergleichsgruppe mit Personen, deren Arbeitgebende den EGZ erhalten hatten. Dieser längere Verbleib im Erwerbsleben könnte auch durch eine leistungssteigernde Wirkung und stärkere Zielorientierung gefördert worden sein, die schon bei anderen Studien über Prämienzahlungen beobachtet wurden 6 7 .

Die bereits erwähnten Interviewanalysen, die im Rahmen dieser Studie durchgeführt wurden ergaben, dass die ESP zumeist für Mobilität, Arbeitskleidung und finanzielle Stabilisierung verwendet wurde. Konkret am Beispiel der Mobilität wurde die ESP u. a. für die Verbesserung des Arbeitswegs durch Monatsfahrkarten und eine Autoteilfinanzierung verwendet 11 . Geht man davon aus, dass diese Anschaffungen als Arbeitsplatzinterventionen deklariert werden können, dann stehen unsere Ergebnisse im Einklang mit Reviews, die zeigten, dass Maßnahmen am Arbeitsplatz wirksam dazu beitragen, Personen wieder in Arbeit zu bringen 12 13 14 . Wir konnten zwar zeigen, dass die Personen der ESP-Gruppe länger erwerbstätig waren, als die Personen in der EGZ-Gruppe, allerdings können wir daraus nicht schließen, dass die ESP generell besser geeignet ist als der EGZ. Vielmehr ist davon auszugehen, dass mit der Wahlmöglichkeit zwischen ESP und EGZ die Passung der Leistung mit den jeweils sehr individuell gelagerten Fällen verbessert wird. Während bspw. eine Person mit starken Einschränkungen ggf. ausschließlich mit der Unterstützung durch den EGZ einen Arbeitsplatz erhält, kann eine andere Person die ESP verwenden, um mit dem Auto pünktlich und flexibel die Arbeitsstelle zu erreichen.

Um eine Überlegenheit der ESP gegenüber dem EGZ zu zeigen, müsste eine randomisierte Zuordnung ohne Wahlmöglichkeit erfolgen. Damit könnten u. a. person- und umweltbezogene Faktoren identifiziert werden, die als Förder- bzw. Hemmfaktoren für eine Rückkehr ins Erwerbsleben eine Rolle spielen können.

Die höhere Chance von Versicherten der DRV BS-H eine 15-monatige Erwerbstätigkeit zu erreichen, könnte neben den unterschiedlichen Arbeitsmarktsituationen unter anderem an zwei unterschiedlichen Beratungsprozessen liegen. Während Versicherte der DRV BS-H mindestens ein Beratungsgespräch erhielten, wurden die Bescheide zur Genehmigung der ESP in der Regel von der DRV MD nach Aktenlage versendet. Möglicherweise besteht in der persönlichen Beratung ein Potential für die Abklärung von Hürden und den Verweis auf andere Leistungen. Eine angemessene Beratung ist ein zielorientierter Prozess, in dem Fachleute Ratsuchende zu Themen der beruflichen Rehabilitation unterstützen. Sie kann Personen mit gesundheitlichen Beeinträchtigungen dabei helfen, neue berufliche Perspektiven zu entdecken 15 . Sternberg et al. zeigen, dass eine gemeinsame Einschätzung von Klient:innen und Fachberatenden erfolgsversprechend ist 16 .

Es konnte gezeigt werden, dass knapp 60% der Teilnehmenden mindestens ein Jahr vor der Intervention nicht versicherungspflichtig beschäftigt waren. Die Frage, ob mit der ESP insbesondere „Langzeitarbeitslose“ adressiert werden konnten, kann allerdings nicht mit Sicherheit beantwortet werden.

### Limitationen

Als Limitation ist zu nennen, dass bei der Interpretation der Ergebnisse beachtet werden muss, dass es sich um eine Wahlleistung handelt und die Teilnehmenden sich jene Maßnahme auswählen konnten, die ihnen mehr zusagte. Damit ist ein Selektionsbias nicht auszuschließen. Zur Berechnung der Maßnahmenkosten wurden im Rahmen der Plausibilitätsprüfung ESP-Kosten aus der Analyse ausgeschlossen, wenn sie den bei der ESP möglichen Höchstwert von 3.000 € überstiegen. Dies trat im Datensatz DRV BS-H auf. Es kann dadurch nicht ausgeschlossen werden, dass immer noch Zahlungen im Datensatz DRV BS-H enthalten sind, die nicht im Kontext der ESP geleistet wurden. Möglicherweise könnte dadurch die Summe der ESP-Kosten von DRV BS-H höher ausfallen, als sie real war. Die Kosten für den ESP-Maßnahmenzeitraum und die ESP-Prämie sind im Datensatz von DRV MD eindeutig identifizierbar, da es sich bei den Maßnahmenkosten stehts um Werte bis 2.400 € bei den ESP-Maßnahmenkosten und 600 € bei den ESP-Prämien handelt. Im Datensatz DRV BSH waren die Werte nicht eindeutig zuzuordnen, daher kann hier keine separate Analyse über ESP-Maßnahmenkosten und ESP-Prämienkosten erfolgen. Unklar bleibt, ob die unterschiedlichen Arbeitslosigkeitsquoten bei Antragstellung mit der Möglichkeit der nachträglichen Bewilligung zusammenhängen. Bei der DRV BS-H war es üblich, dass die ESP z. T. 2 Monate rückwirkend bewilligt wurde. Hierbei ist unklar, welche Arbeit vor dem Antrag von den Versicherten angegeben worden ist: ob es sich um die neue Arbeitsstelle handelt oder um die Arbeitsstelle vor Bezug der ESP. Mit der nachträglichen Bewilligung wollte die DRV ihren Versicherten die Wahl zwischen ESP und EGZ offenhalten, um die Arbeitsaufnahme nicht zu gefährden, wenn Arbeitgebende nicht auf den EGZ verzichten wollten.

Der Interventionszeitraum lag im Zeitraum der COVID-19-Pandemie. Wir können nur spekulieren, welche Auswirkungen dies auf die Studie hatte, da wir keine systematischen Daten dazu erfasst haben. Allgemein war diese Zeit von unsicheren Arbeitsplätzen z. B. in der Gastronomie geprägt und von sozialer Isolation. Möglicherweise konnte die ESP in diesen Fällen neue Optionen ermöglichen und berufliche Teilhabe zu fördern durch die freie Wahl zwischen ESP und EGZ. Im Studienverlauf führte die Pandemie zu einer verzögerten Rekrutierung für die Interviews.

Eine Stärke der Studie ist, dass sie in einem realistischen Setting getestet wurde, in dem bewusst auf Selbstselektion gesetzt wurde. Des Weiteren handelt es sich um eine Vollerhebung. Die hier dargestellten Ergebnisse sind damit relevant für eine mögliche Verstetigung.

## Kernbotschaft

Die Einstiegsprämie ist eine nicht zweckgebundene Leistung an Arbeitnehmende. Im Vergleich zu Zahlungen an Arbeitgebende erzielt sie als Wahlalternative eine sehr gute Eingliederungsquote bei niedrigeren Kosten. Sie zeichnet sich durch besonders gute Ergebnisse für langfristige Beschäftigungsverhältnisse aus.

## Fördermittel

Bundesministerium für Arbeit und Soziales — Fördernummer: 1S013465030P1

## Zusätzliches Material

Zusätzliches Material Zusätzliches Material
