# Supplementary material for: Successful reintegration and economic viability: The entry bonus as a new measure for returning to work
Source: Rehabilitation (Stuttg). 2025 Dec 9;65(5):313–9. [Article in German] doi: 10.1055/a-2724-3608 (PMC13686778; doi:10.1055/a-2724-3608)
Supplement: Supplementary file 1 [file 10-1055-a-2724-3608-2025-05-0037-oa.pdf]

Zusatzmaterial

Tabelle S1: Outcomeparameter, die Datenquellen, Feldbezeichnungen und statistische Analysen.

Outcome	Datenquelle	Statistische Analyse
Soziodemografische Daten<br>Geschlecht und Geburtsjahr	RSD Feldbezeichnung GE<br>Erläuterung: Geschlecht<br>1 = männlich<br>2 = weiblich<br>3 = ohne Eintrag / unbestimmt<br>4 = divers<br>Feldbezeichnung GBJA<br>Erläuterung: Geburtsjahr des Antragstellers	Mittelwerte, Standardabweichungen, Minimal- und Maximalwerte, Anzahl und relative Anzahl
Dauer sozialversicherungspflichtiger Beschäftigung	RSD Feldbezeichnung BY<br>Erläuterung: Beiträge<br>20 = Pflichtbeitragszeit aufgrund versicherungspflichtiger Beschäftigung im ursprünglichen Bundesgebiet (einschließlich Lohnfortzahlung, Nachversicherung nach § 8 SGB VI, Gleitzonenmischfälle und Fälle in denen der Arbeitnehmer auf die Anwendung der Gleitzonenregelung verzichtet (§ 163 Abs. 10 SGB VI); hierunter fallen nicht Beitragszeiten aufgrund einer Berufsausbildung, nach dem Altersteilzeitgesetz und Beitragszeiten mit reinen Entgelten in der Gleitzone)<br>25 = Pflichtbeitragszeit aufgrund versicherungspflichtiger Beschäftigung in den neuen Ländern einschließlich des Ostteils Berlins (nähere Erläuterung siehe Schlüsselziffer 20)<br>38 = Pflichtbeitragszeit aufgrund eines Beschäftigungsverhältnis mit reinem Entgelt in der Gleitzone (§ 20 Abs. 2 SGB IV) (ohne Gleitzonenmischfälle und Fälle in denen der Arbeitnehmer auf die Anwendung der Gleitzonenregelung verzichtet (§ 163 Abs. 10 SGB VI)).<br>71 = Pflichtbeitragszeit wegen Leistungsbezug von Arbeitslosengeld<br>85 = Geringfügige Beschäftigung (mit und ohne Befreiung von der Versicherungspflicht, bzw. mit und ohne Verzicht auf die	Summen, Anzahl und relative Anzahl

	Versicherungsfreiheit (bis 31.12.2012); auch im Privathaushalt) (§ 8 Abs. 1 Nr. 1 SGB IV, § 8a SGB IV)	
Maßnahmenkosten	Zahldaten der DRVen MD und BS-H	Summen, Mittelwerte, Standardabweichung, Minimum- und Maximalwerte, Spannweite
Beitragszeiten 12 Monate vor Maßnahmenbeginn	RSD Feldbezeichnung BY<br>Erläuterung: Beiträge<br>00 = keine freiwilligen oder Pflichtbeiträge<br>10 = freiwillige Beitragszeit<br>20 = Pflichtbeitragszeit aufgrund versicherungspflichtiger Beschäftigung im ursprünglichen Bundesgebiet (einschließlich Lohnfortzahlung, Nachversicherung nach § 8 SGB VI, Gleitzonenmischfälle und Fälle in denen der Arbeitnehmer auf die Anwendung der Gleitzonenregelung verzichtet (§ 163 Abs. 10 SGB VI); hierunter fallen nicht Beitragszeiten aufgrund einer Berufsausbildung, nach dem Altersteilzeitgesetz und Beitragszeiten mit reinen Entgelten in der Gleitzone)<br>25 = Pflichtbeitragszeit aufgrund versicherungspflichtiger Beschäftigung in den neuen Ländern einschließlich des Ostteils Berlins (nähere Erläuterung siehe Schlüsselziffer 20)<br>71 = Pflichtbeitragszeit wegen Leistungsbezug von Arbeitslosengeld<br>85 = Geringfügige Beschäftigung (mit und ohne Befreiung von der Versicherungspflicht, bzw. mit und ohne Verzicht auf die Versicherungsfreiheit (bis 31.12.2012); auch im Privathaushalt) (§ 8 Abs. 1 Nr. 1 SGB IV, § 8a SGB IV)	Anzahl, relative Anzahl, Mittelwerte, Standardabweichung
Abbrüche	Statistik 96<br>Feldbezeichnung EWMS<br>Erläuterung: Ergebnis der Leistung<br>00 = keine Angabe<br>05 = Prüfung nicht bestanden	Anzahl und relative Anzahl

	10 = mit Erfolg abgeschlossen (staatlich anerkannt)<br>20 = mit Erfolg abgeschlossen (Zeugnis der Ausbildungsstätte oder Leistung ohne Prüfung und Zeugnis; auch WfbM)<br>Abbruch durch den Versicherten vorwiegend<br>30 = wegen Aufnahme einer Beschäftigung/Tätigkeit<br>40 = aus leistungsmäßigen Gründen<br>50 = aus gesundheitlichen Gründen<br>60 = aus wirtschaftlichen Gründen<br>70 = aus persönlichen Gründen<br>90 = aus sonstigen Gründen<br>Abbruch durch den Versicherungsträger vorwiegend<br>45 = aus leistungsmäßigen Gründen<br>85 = aus disziplinarischen Gründen<br>95 = aus sonstigen Gründen	
Berufliche Stellung	Statistik 96<br>Feldbezeichnung STBF<br>Erläuterung: Stellung im Beruf/Erwerbsleben<br>0 = nicht erwerbstätig (z. B. Hausfrau, nicht erwerbstätiger Rentner, Vorruhestandsgeldempfänger)<br>1 = Auszubildender (Lehrling, Anlernling, Praktikant, Volontär, Student)<br>2 = ungelernter Arbeiter (nicht als Facharbeiter tätig)<br>3 = angelernter Arbeiter in anerkanntem Anlernberuf (nicht als Facharbeiter tätig)<br>4 = Facharbeiter<br>5 = Meister, Polier (gleichgültig ob Arbeiter oder Angestellter)<br>6 = Angestellter (aber nicht Meister, Polier im Angestelltenverhältnis)<br>7 = Beamter/DO-Angestellter, Versorgungsempfänger i. S. d. Beamtenrechtes<br>8 = Selbständiger	Anzahl und relative Anzahl
Berufliche Tätigkeit bei Antragsstellung	Statistik 96<br>Feldbezeichnung AIAQ<br>Erläuterung: Arbeit vor Antragstellung<br>0 = nicht erwerbstätig, aber nicht 6 oder 7<br>1 = Ganztagsarbeit ohne Wechselschicht/Akkord/Nachtschicht	Anzahl und relative Anzahl

	2 = Ganztagsarbeit mit Wechselschicht/Akkord<br>3 = Ganztagsarbeit mit Nachtschicht<br>4 = Teilzeitarbeit weniger als die Hälfte der üblichen Arbeitszeit<br>5 = Teilzeitarbeit mindestens die Hälfte der üblichen Arbeitszeit<br>6 = ausschließlich Tätigkeit als Hausfrau/Hausmann<br>7 = arbeitslos gemeldet<br>8 = Heimarbeit<br>9 = Beschäftigung in einer Werkstatt für behinderte Menschen	
Branchen	Statistik 96<br>Feldbezeichnung: BFKL<br>Erläuterung: Zuletzt ausgeübte Tätigkeit	Anzahl und relative Anzahl
Maßnahmenbeginn und Ende	Statistik 96<br>Feldbezeichnung MSVN<br>Erläuterung: Beginn der Leistung<br>Feldbezeichnung MSBS<br>Erläuterung: Ende der Maßnahme	Daten stellten den Start- und Endpunkt der Zählung der Beitragszeiten vor und nach der Intervention dar.

Tabelle S2: Häufigkeiten der Branchen, in denen die Teilnehmenden tätig waren.

**Branchen**	**Gesamtkohorte**			**DRV Mitteldeutschland (MD)**		
n (%)	Gesamt (n=2204)	ESP (n=485)	EGZ (n=1719)	Gesamt (n=889)	ESP (n=224)	EGZ (n=665)
Militär	14 (0,6)	2 (0,4)	12 (0,7)	6 (0,7)	2 (0,9)	4 (0,6)
Land-, Forst-, Tierwirtschaft, Gartenbau	79 (3,6)	12 (2,5)	67 (3,9)	31 (3,5)	5 (2,2)	26 (3,9)
Rohstoffgewinnung, Produktion, Fertigung	748 (33,9)	145 (29,9)	603 (35,1)	326 (36,7)	82 (36,6)	244 (36,7)
Bau, Architektur, Vermessung, Gebäudetechnik	378 (17,2)	70 (14,4)	308 (17,9)	166 (18,7)	39 (17,4)	127 (19,1)
Naturwissenschaft, Geografie, Informatik	26 (1,2)	8 1,6)	18 (1,0)	12 (1,3)	4 (1,8)	8 (1,2)
Verkehr, Logistik, Schutz und Sicherheit	453 (20,6)	101 (20,8)	352 (20,5)	160 (18,0)	36 (16,1)	124 (18,6)
Kaufm. Dienstl., Handel, Vertrieb, Tourismus	188 (8,5)	51 (10,5)	137 (8,0)	73 (8,2)	23 (10,3)	50 (7,5)
Unternehmensorga, Buchhalt, Recht, Verwalt.	82 (3,7)	27 (5,6)	55 (3,2)	25 (2,8)	11 (4,9)	14 (2,1)
Gesundheit, Soziales, Lehre u. Erziehung	213 (9,7)	60 (12,4)	153 (8,9)	83 (0,3)	19 (8,9)	64 (9,6)
Geisteswissenschaften, Kultur, Gestaltung	23 (1,0)	9 (1,9)	14 (0,8)	7 (0,8)	3 (1,3)	4 (0,6)

Tabelle S3: Häufigkeiten der Stellung im Beruf in der Gesamtkohorte und separat in den DRVen MD und BS-H.

**Stellung im Beruf**	**Gesamtkohorte n (%)**			**DRV Mitteldeutschland (MD) n (%)**			**DRV Braunschweig-Hannover (BS-H) n(%)**		
	Gesamt (n=2204)	ESP (n=485)	EGZ (n=1719)	Gesamt (n=889)	ESP (n=224)	EGZ (n=665)	Gesamt (n=1.315)	ESP (n=261)	EGZ (n=1054)
nicht erwerbstätig	12 (0,5)	3 (0,6)	9 (0,5)	3 (0,3)	1 (0,4)	2 (0,3)	9 (0,7)	2 (0,8)	7 (0,7)
Auszubildende:r	9 (0,4)	2 (0,4)	7 (0,4)	2 (0,2)	0 (0)	2 (0,3)	7 (0,5)	2 (0,8)	5 (0,5)
ungelernte:r Arbeiter:in	170 (7,7)	35 (7,2)	135 (7,8)	68 (7,6)	19 (8,5)	49 (7,4)	102 (7,8)	16 (6,1)	86 (8,2)
angelernte:r Arbeiter:in	200 (9,1)	47 (9,7)	153 (8,9)	83 (9,3)	26 (11,6)	57 (8,6)	117 (8,9)	21 (8,0)	96 (9,1)
Facharbeiter:in	1.279 (58,0)	278 (57,3)	1.001 (58,2)	571 (64,2)	143 (63,8)	428 (64,4)	708 (53,8)	135 (51,7)	573 (54,4)
Meister, Polier	34 (1,5)	7 (1,4)	27 (1,6)	11 (1,2)	3 (1,3)	8 (1,2)	23 (1,7)	4 (1,5)	19 (1,8)
Angestellte:r	491 (22,3)	111 (22,9)	380 (22,1)	150 (16,9)	31 (13,8)	119 (17,9)	341 (25,9)	80 (30,7)	261 (24,8)
Selbstständige:r	9 (0,4)	2 (0,4)	7 (0,4)	1 (0,1)	1 (0,4)	0 (0)	8 (0,6)	1 (0,4)	7 (0,7)

Tabelle S4: Häufigkeiten der Arbeit bei Antragsstellung in MD und BS-H.

**Arbeit vor Antrag**	**DRV Mitteldeutschland (MD)**		**DRV Braunschweig-Hannover (BS-H)**	
	n	%	n	%
nicht erwerbstätig	4	0,4	15	1,1
Ganztagsarbeit ohne Wechselschicht	275	30,9	822	62,5
Ganztagsarbeit mit Wechselschicht	91	10,2	121	9,2
Ganztagsarbeit mit Nachtschicht	46	5,2	69	5,2
Teilzeitarbeit <50%	3	0,3	15	1,1
Teilzeitarbeit >=50%	53	6,0	92	7,0
ausschließlich Tätigkeit als Hausmann/Hausfrau	n.a.	n.a.	3	0,2
arbeitslos gemeldet	417	46,9	178	13,5

Tabelle S5: Ergebnisse der binär logistischen Regressionsanalyse zur 15-monatigen Erwerbstätigkeit (n=2.204) (*df: Freiheitsgrad, Ref: Referenzkategorie).*

	Regressions-koeffizient B	Standardfehler	Wald	df	*p*	Exp(B)	95% Konfidenzintervall	
							Unterer Wert	Oberer Wert
ESP oder EGZ (Ref. EGZ)	0,868	0,127	46,409	1	< 0,001	2,383	1,856	3,059
Alter bei Maßnahmenbeginn	-0,022	0,006	15,312	1	< 0,000	0,978	0,967	0,989
Trägerin DRV (Ref. MD)	0,361	0,096	14,130	1	< 0,001	1,435	1,189	1,732
